# Supplementary material for: Implications of monocular vision for racing drivers
Source: PLoS One. 2019 Dec 16;14(12):e0226308. doi: 10.1371/journal.pone.0226308 (PMC6913915; doi:10.1371/journal.pone.0226308)
Supplement: Attachment — Submitted filename: Response to reviewers.docx [file pone.0226308.s009.docx]

**Response to reviewers**

November 2019

In red are the response of the authors

**Responses to Reviewer #2:**

Reviewer #2: (1) It may be worth pointing out that the ANOVA is for the repeated measurements.

Answer:

We appreciate the reviewer’s question. we have modified:

Page 9 line 196-197 “We also analyzed driver reaction time in crash avoidance using two-way analysis of variances (ANOVAs) with repeated measures.” by “We also analyzed driver reaction time in crash avoidance using two-way repeated measures ANOVA.

Page 13 line 282-283 “The two-way ANOVA with repeated measures showed significant main effects of the visual condition” by “The two-way repeated measures ANOVA showed significant main effects of the visual condition”.

(2) The authors found that there is no strong correlation between the reaction time and the accident rate in the baseline condition, and argued that "this can be explained by the fact that in baseline conditions, the accident relies more on the intrinsic qualities of each driver to be able to avoid the accident." I wonder if the authors can provide any evidence to support their claim.

We appreciate the reviewer’s question. There is no specific evidence we are currently aware of and this claim is only made based on our analysis and observations. We should have used instead :“this **may** be explained by the fact that in baseline conditions, the accident relies more on the intrinsic qualities of each driver to be able to avoid the accident. If the reviewer is aware of any evidence we would be open to including it here.
